# STAT3 Post-Translational Modifications Drive Cellular Signaling Pathways in Prostate Cancer Cells

**DOI:** 10.3390/ijms20081815

**Published:** 2019-04-12

**Authors:** Rossana Cocchiola, Elisabetta Rubini, Fabio Altieri, Silvia Chichiarelli, Giuliano Paglia, Donatella Romaniello, Stefania Carissimi, Alessandra Giorgi, Flavia Giamogante, Alberto Macone, Giacomo Perugia, Aymone Gurtner, Margherita Eufemi

**Affiliations:** 1 Department of Biochemical Sciences “A. Rossi Fanelli” and Istituto Pasteur-Fondazione Cenci Bolognetti, Sapienza University, P.le A. Moro 5, 00185 Rome, Italy; rossana.cocchiola@uniroma1.it (R.C.); elisabetta.rubini@uniroma1.it (E.R.); fabio.altieri@uniroma1.it (F.A.); silvia.chichiarelli@uniroma1.it (S.C.); giuliano.paglia@uniroma1.it (G.P.); stefania.carissimi@uniroma1.it (S.C.); alessandra.giorgi@uniroma1.it (A.G.); flavia.giamogante@uniroma1.it (F.G.); alberto.macone@uniroma1.it (A.M.); 2 Department of Biological Regulation, Weizmann Institute of Science, 234 Herzl Street, 7610001 Rehovot, Israel; donatella.romaniello@weizmann.ac.il; 3 Department of Gynecological-Obstretic Science and Urologic Sciences, Sapienza University, V.le Dell’Università, 00185 Rome, Italy; giacomo.perugia@uniroma1.it; 4 Department of Research, Advanced Diagnostics, and Technological Innovation, Translational Research Area, Regina Elena National Cancer Institute; via Elio Chianesi, 53, 00144 Rome, Italy; aymone.gurtner@ifo.gov.it

**Keywords:** STAT3, post translational modification, prostate cancer, transduction signaling

## Abstract

STAT3 is an oncoprotein overexpressed in different types of tumors, including prostate cancer (PCa), and its activity is modulated by a variety of post-translational modifications (PTMs). Prostate cancer represents the most common cancer diagnosed in men, and each phase of tumor progression displays specific cellular conditions: inflammation is predominant in tumor’s early stage, whereas oxidative stress is typical of clinically advanced PCa. The aim of this research is to assess the correspondence between the stimulus-specificity of STAT3 PTMs and definite STAT3-mediated transcriptional programs, in order to identify new suitable pharmacological targets for PCa treatment. Experiments were performed on less-aggressive LNCaP and more aggressive DU-145 cell lines, simulating inflammatory and oxidative-stress conditions. Cellular studies confirmed pY705-STAT3 as common denominator of all STAT3-mediated signaling. In addition, acK685-STAT3 was found in response to IL-6, whereas glutC328/542-STAT3 and pS727-STAT3 occurred upon tert-butyl hydroperoxyde (tBHP) treatment. Obtained results also provided evidence of an interplay between STAT3 PTMs and specific protein interactors such as P300 and APE1/Ref-1. In accordance with these outcomes, mRNA levels of STAT3-target genes seemed to follow the differing STAT3 PTMs. These results highlighted the role of STAT3 and its PTMs as drivers in the progression of PCa.

## 1. Introduction

Prostate cancer (PCa) represents one of the leading causes of morbidity and mortality among adult males in Western countries. It is a multifactorial and biologically heterogeneous disease that could progress to an advanced hormone-refractory stage, which is still considered incurable [1,2].

Prostate cancer is characterized by the dysregulation of several intracellular pathways and its onset and progression are determined by the presence and activation status of the androgen receptor (AR), which plays a key role during all phases of tumor growth. In fact, the AR-signaling axis not only regulates normal prostate cell morphogenesis and differentiation, but is also involved in the transition from androgen-dependent PCa to a more aggressive phenotype [3]. Although AR constitutes the main character in cancer development, a number of AR-mediated pathways were found to interact with other intracellular networks that are critically implicated in the PCa setting, especially in advanced phases. These pathways include growth factor receptor signaling, MAPK signaling and cytokine signaling [4,5,6,7].

Several in vitro and in vivo studies identify STAT3 (signal transducer and activator of transcription 3) as a main player in cellular events responsible for the insurgence, progression [8,9] and development of metastatic PCa [10,11,12].

STAT3 is a key signaling protein with different biological effects ranging from cellular growth to differentiation and survival in response to different stimuli such as growth factors (EGF, TGF-α) [13], cytokines (IL-6, INF-α) [14] or cytosolic kinases (Src, Tyk2, Abl) [15].

STAT3 can fulfil its multifaceted biological activities through two main intracellular signaling patterns known as the canonical and the non-canonical pathways of STAT3 [16]. In the canonical pathway, STAT3 phosphorylation at Y705 drives its oncogenic activity through its homodimerization and nuclear translocation. As a transcription factor, STAT3 directly regulates the expression of a wide range of genes, many of which play key roles in oncogenesis. The non-canonical pathway embraces several STAT3 functions that have been shown to be independent of its phosphorylation at Y705 [17]. These biological activities are summarized below:

- un-phosphorylated STAT3 (U-STAT3) can bind to the interferon γ-activated sequence (GAS), either as dimer or as monomer. The U-STAT3 regulates gene expression via its binding to AT-rich DNA sequences and drives chromatin structure remodeling [18];
- STAT3 phosphorylation at S727, rather than Y705, is required for its mitochondrial activity [19]. In particular, has been reported that mitochondrial STAT3 controls cell respiration and metabolism by enhancing the activity of succinate oxidoreductase (complex II), ATP synthase (complex V) and lactate dehydrogenase. Affecting the balance between glycolytic and oxidative phosphorylation metabolisms, STAT3 provides the necessary requirements to support the metabolic shift toward aerobic glycolysis known as the Warburg effect [20];
- STAT3 was found associated with a variety of cytosolic structures, including focal adhesions, microtubules and mitotic spindles. Indeed, STAT3 plays an important role during the assembly of cytoskeleton networks, such as the actin filaments and microtubules, thus promoting cell migration and invasion [21].

The biological consequences of STAT3 regulation and activation are quite complex. Aside from its phosphorylation on Y705, STAT3 presents a wide variety of post-translational modifications (PTMs) that affect STAT3 nuclear function other than its non-genomic activities. In fact, STAT3 was also found phosphorylated on S727, acetylated on K685 and glutathionylated on Cys residues 328 and 542; each PTM is involved in a specific STAT3 activity [22].

The phosphorylation on S727, as well as the S-gluthathionylation on C328 and C542, occurs under oxidative-stress conditions [23,24]. In particular, pS727-STAT3 seems to be essential for mitochondrial STAT3 activity, while STAT3 S-glutathionylation prevents STAT3 phosphorylation on Y705 and inhibits the formation of STAT3 dimers, precluding STAT3 canonical transcriptional activity.

On the other hand, cytokines stimuli induce STAT3 acetylation, and the balance between the protein acetylation/deacetylation is a molecular switch that controls the expression of inflammatory genes [25].

In recent years, several studies have established that the post-translational modifications of proteins address the flow of molecular information from the extracellular environment to intracellular compartments via the activation of specific signaling pathways. Chemical modifications of key proteins involved in signal transduction could regulate numerous physiological events, such as gene transcription, cell proliferation, differentiation and apoptosis, tissue development and tumor progression [26]. STAT3 represents a hub protein in the intricate network of intracellular pathways and its different PTMs (p-Y705, p-S727, Ac-K685 and Glut-C328/C542) orchestrate its pleiotropic activity, reflecting specific cellular states as inflammation or oxidative stress [27].

On the basis of literature data, together with our results from previous studies on prostate cancer FFPE tissues [28], it is conceivable to hypothesize that STAT3 and its PTMs (which are observable in different PCa clinical stages, as measured by Gleason score grading) may be involved in the progression of PCa into a more aggressive form. Taking into account all these evidences, the aim of our study is to investigate the relationship among STAT3 activation (p-Y705) and other STAT3 PTMs (p-S727, Ac-K685 and Glut-C328/542) in the progression and aggressiveness of human PCa. To do this, in vitro studies were performed on less aggressive LNCaP, and more-aggressive DU-145 prostate cancer cell lines were subjected to different stimuli.

## 2. Results

### 2.1. STAT3 Post-Translational Modifications (PTMs) Modulate Different Signal Transduction PATHWAYS in PCa

**Figure 1:**
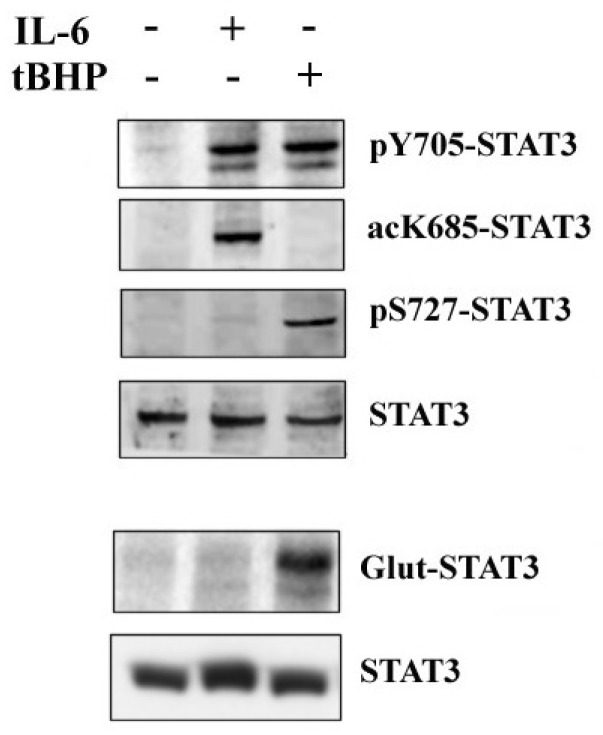
Western blotting analysis of STAT3 PTMs. The STAT3 PTMs, p-Y705, pS-727 and ac-K685, are detected by immunoblotting of cellular extracts obtained from LNCaP cells both untreated and treated with IL-6 or tBHP. Glut-STAT3 was detected on samples immunoprecipitated with anti-STAT3 antibody. Samples, both untreated and treated with IL-6 or tBHP, were first separated by SDS-PAGE under nonreducing conditions, then subjected to western blot analysis using monoclonal anti-glutathione and anti-STAT3 antibodies.

PCa is a multifactorial disease, usually associated with chronic prostatic inflammation. Sustained inflammatory conditions can lead to generation of free radicals that can be responsible for PCa progression and transformation into a more aggressive form. Chronic inflammation and oxidative stress represent closely connected and related processes that do not exclude each other [29], but it can be supposed that chronic inflammation is mainly characteristic of early PCa stages [30], while oxidative stress is a more typical condition of tumors at advanced clinical phases [31]. Previous studies on prostate cancer FFPE tissues highlighted a different STAT3 PTM pattern in PCa specimens at different clinical stages [28], so we hypothesize that STAT3 PTMs may be involved in PCa progression, and can modulate different signal transduction pathways depending on the triggering stimuli. Thus, LNCaP were treated with IL-6 to induce an inflammatory response, whereas tert-butyl hydroperoxyde (tBHP) was used to simulate oxidative stress. The expression profile of STAT3 PTMs was analyzed by western blot in both untreated and treated LNCaP cells (Figure 1). The results confirmed that STAT3 phosphorylation at Y705 is a common PTM involved in all STAT3 activation pathways, whilst other modifications could be defined as “stimulus-specific”. In fact, STAT3 acetylation at K685 is typical of the cytokinin response, and the phosphorylation on S727 or glutathionylation on C328/C542 are present after tBHP stimulation.

### 2.2. STAT3 PTMs: Cellular Condition Sensors and Trascriptional Program Drivers

In order to understand if the stimulus-dependent STAT3 PTMs could be a link between signal transduction and specific cellular conditions, their correspondence with definite STAT3-mediated transcriptional programs should be investigated. Only results from samples treated with tBHP will be reported, but analogous outcomes were also obtained with hydrogen peroxide (data not showed).

ChIP and RT-qPCR experiments were performed on LNCaP cells treated with IL-6 and tBHP, testing STAT3-specific genes: c-reactive protein (*CRP*) representing inflammation [32,33], superoxide dismutase 2 (*SOD2*) [34,35] and matrix metalloproteinase-2 (*MMP2*) [36,37] for oxidative stress and survivin (*BIRC5*) representing both states [38]. Genes were selected based on previous results on prostate cancer FFPE tissues at different Gleason scores [28].

**Figure 2:**
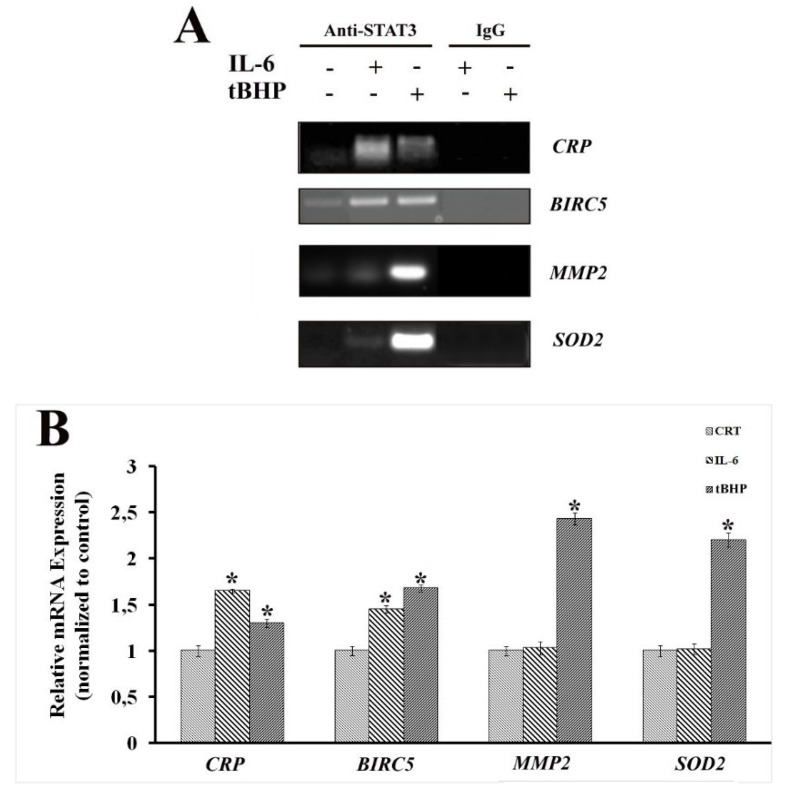
(**A**) PCR analysis of DNA fragments from chromatin immunoprecipitation (ChIP) performed with anti-STAT3 antibody. Enrichment of specific STAT3-binding sites present in the immunoprecipitated DNA fragments from LNCaP both untreated and treated with IL-6 or tBHP. (**B**) Real-time PCR analysis of control immunoprecipitation performed with pre-immune IgGs is also reported. Quantification of mRNA expression relative to STAT3-specific genes is representative of the inflammation state and oxidative stress in LNCaP cells. Results are the average of three independent measurements. Statistically significant differences (*p* < 0.05) between samples both untreated and treated with IL-6 or tBHP are marked by *. (CRT: untreated control).

The outcomes showed that STAT3-DNA binding activity in the promoter region of tested genes was stimulus-specific and, consequently, related to STAT3 PTMs. In fact, samples treated with IL-6 exhibited enrichment mainly for *CRP* promoter, whereas an increased binding to *SOD2* and *MMP2* promoters was found after tBHP stimulation. STAT3 binding to *BIRC5* promoter was detected after both IL-6 and tBHP treatment (Figure 2A). To further confirm the role of stimulus-specific PTMs as guiding lights for STAT3 transcriptional program, the profile expression for the same genes was evaluated by RT-qPCR analysis. Figure 2B clearly shows that *SOD2* and *MMP2* are overexpressed only upon oxidative stress. Although *CRP* and *BIRC5* were overexpressed in both conditions, confirming the close interconnection between chronic inflammation and oxidative stress, a difference in the response can be observed. In fact, *CRP* overexpression seems prominent following the IL-6 treatment, while *BIRC5* seems more related to the tBHP treatment.

### 2.3. STAT3 Partners: PTM Modulators

All STAT3 PTMs reflect the dynamic balance between epigenetic “writers” and “erasers”. In fact, STAT3 can fulfil the multitude of its intracellular functions collaborating with specific protein partners [39]. To identify the potential modulators of STAT3 PTMs, co-immunoprecipitation (CoIP) was performed in LNCaP cells under the same experimental conditions as the above-mentioned ChIP assay.

**Figure 3:**
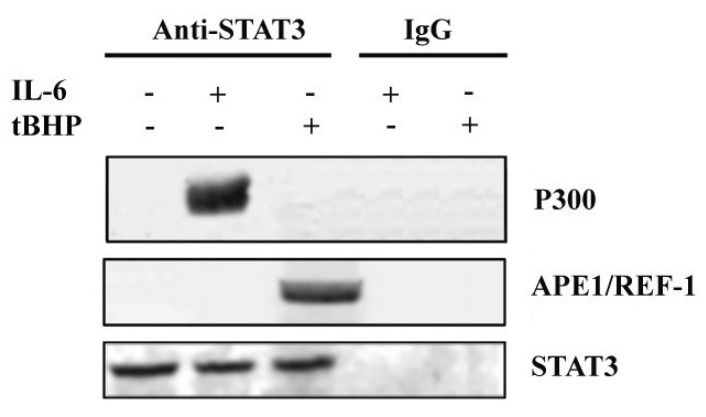
Co-immunoprecipitation (CoIP) and western blotting analysis of the co-immunoprecipitated proteins with anti-STAT3 antibody. The CoIP was performed on LNCaP cells both untreated and treated with IL-6 or tBHP. Pre-immune IgGs were used as the control.

A preliminary analysis of immunocomplexes by mass spectrometry (data not shown) allowed us to identify several STAT3 co-immunoprecipitated proteins. Among the identified proteins, a particular attention was focused on P300 and APE1/Ref-1, which have been previously reported as STAT3-specific interactors [40,41]. The protein P300 is a histone acetyltransferase (HAT) typically recruited by transcriptional enhancers, and regulates gene expression [42]. APE1/Ref-1 is a multifunctional protein possessing both DNA repair and transcriptional regulatory activities, and has a pleiotropic role in controlling cellular response to oxidative stress [43]. STAT3 interaction with P300 and APE1/Ref-1 was confirmed by western blot analysis of immunocomplexes using specific antibodies. Figure 3 shows the presence of P300 protein in the STAT3-immunoprecipitated complexes obtained from IL-6 stimulated LNCaP cells, whereas APE1/Ref-1 was co-immunoprecipitated with STAT3 in cells treated with tBHP. These results are in agreement with the specificity of STAT3 PTMs in response to different stimuli.

**Figure 4:**
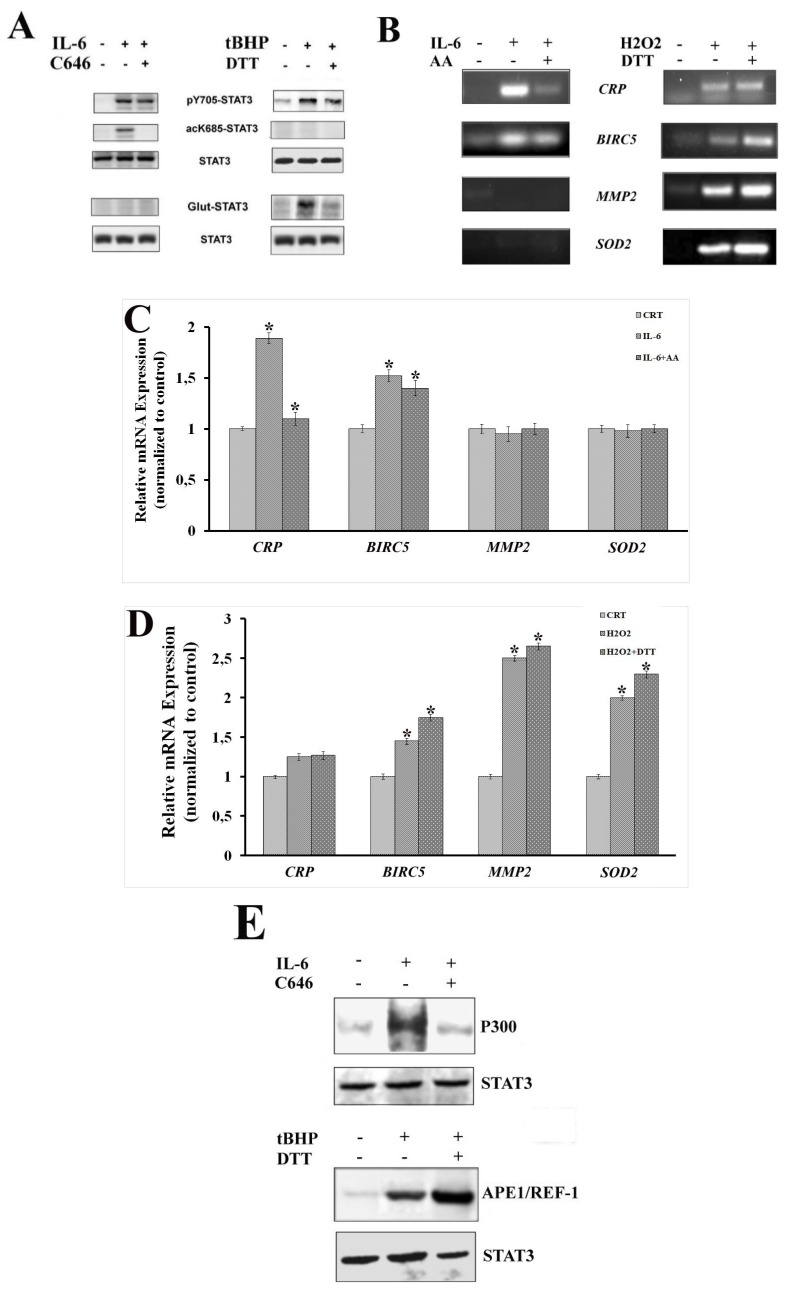
Effect of C646 and dithiothreitol (DTT) on STAT3 PTM profile and STAT3 transcriptional program. (**A**) Western blotting analysis of STAT3 PTMs (p-Y705, p-S727, acK685, in LNCaP cells both untreated and treated with IL-6 and tBHP in the presence of different inhibitors. Glut-STAT3 was detected on samples immunoprecipitated with anti-STAT3 antibody. Samples, both untreated and treated with IL-6 or tBHP in the presence or not of different inhibitors, were first separated by SDS-PAGE under nonreducing conditions, then subjected to western blot analysis using monoclonal anti-glutathione and anti-STAT3 antibodies. (**B**) PCR analysis of specific STAT3-binding sites on immunoprecipitated DNA fragments (ChIP assay) obtained from LNCaP cells both untreated and treated with IL-6 and tBHP in the presence of different inhibitors. (**C**) Real-time PCR analysis and relative expression of STAT3-target genes in LNCaP cells both untreated and treated with IL-6 in the presence of C646. (**D**) Real-time PCR analysis and relative expression of STAT3-target genes in LNCaP cells both untreated and treated with tBHP in the presence of DTT. Statistically significant differences (*p* < 0.05) between samples untreated or treated with IL-6 or tBHP are marked by *, while statistically significant differences (*p* < 0.05) between samples treated with IL-6 or tBHP, either pre-incubated with a specific STAT3 inhibitor or not, are marked by §. (**E**) Western blotting analysis of anti-STAT3 co-immunoprecipitated proteins (CoIP assay) obtained from LNCaP cells both untreated and treated with IL-6 or tBHP in presence of the different inhibitors. (CRT: untreated control).

In the absence of specific inhibitors for STAT3 K685 acetylation and C328 and C542 glutathionylation, to support our findings, we used a P300 inhibitor (C646) which prevented acetylation [44] and dithiothreitol (DTT) as a broad glutathionylation suppressant [45,46]. DTT was selected, among other inhibitors, for its capability to restore the transcriptional function of dimeric STAT3 by reverting STAT3 glutathionylation. Considering the large number of kinases that could be responsible for the phosphorylation of STAT3 at S727, it was hard to find a specific inhibitor for this PTM [47,48,49]. Currently a selective inhibitor for pS727 has not been identified [50]. Thus, western blotting, ChIP, CoIP and RT-qPCR analyses were performed on LNCaP cells in the presence (or not) of the above-mentioned compounds to confirm the influence of intracellular environment on STAT3 PTMs (Figure 4).

As visualized by western blot analysis, STAT3 glutathionylation occurred after tBHP treatment, but the glutathionylation signal disappeared when cells were pre-treated with DTT. Similarly, STAT3 acetylation was evident in LNCaP cells stimulated with IL-6, but not after C646 treatment (Figure 4A).

**Figure 5:**
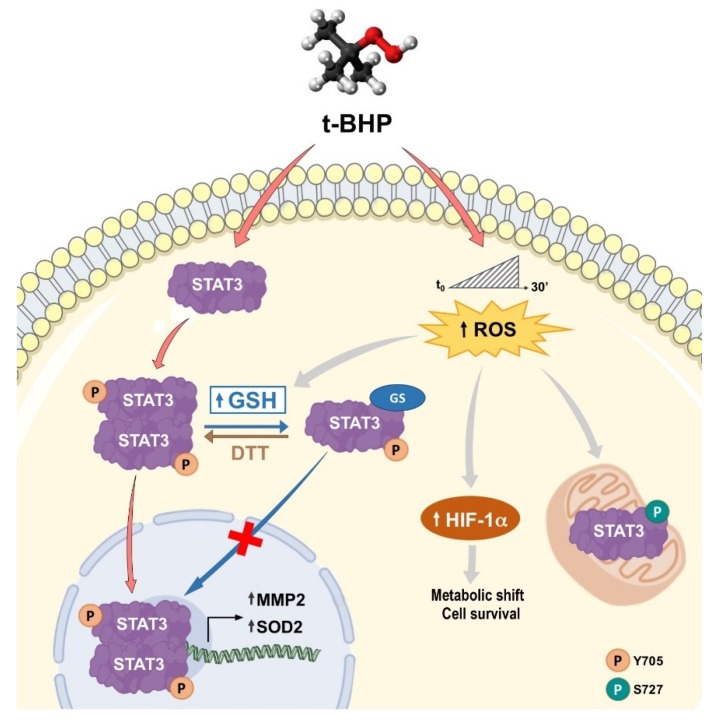
STAT3 PTMs drive the transcriptional program of STAT3 in PCa.

These findings were consolidated by ChIP and RT-qPCR analyses carried out under the same experimental settings. In fact, both STAT3-DNA binding activity and STAT3 transcriptional function seemed to be influenced by inhibition of its specific PTMs in the context of inflammation or oxidative stress (Figure 4B,C). However, the use of DTT as a glutathionylation suppressant seemed unable to reduce *BIRC5*, *SOD2* and *MMP2* expression levels, which were increased by the oxidative stress induced by tBHP treatment. The use of DTT to revert STAT3 glutathionylation can probably reactivate STAT3 and its transcriptional activity (Figure 5).

This is also consistent with a slightly higher expression of *BIRC5*, *SOD2* and *MMP2* when cells were treated with DTT after oxidative-stress induction.

The use of C646 and DTT also affected the composition of STAT3-specific immunocomplexes (Figure 4E). Indeed, in the CoIP assay, the P300 was no more detectable after the treatment with C646. On the other hand, the presence of DTT after oxidative-stress induction increased the amount of STAT3 co-immunoprecipitated Ref-1, probably because in the reduced form both proteins are more capable to carry out their transcription and coactivator functions.

Results obtained from the use of STAT3 acetylation and glutathionylation inhibitors confirmed the stimulus-specificity of these PTMs, as well as their major role in the regulation of STAT3-transcriptional program.

STAT3 phosphorylation at serine residue 727 was been identified as a sensor of ROS generation and hypoxic condition, in addition to driving STAT3 mitochondrial localization [23]. Since a selective inhibitor for p-S727 is not yet available, the correlation between this PTM and the non-canonical STAT3 pathway has been investigated through a study on STAT3 cellular distribution. Moreover, since STAT3 is regulating energy metabolism through the direct interplay with HIF-1α [51,52], an analysis of HIF-1α expression levels was also performed. The protein HIF-1α was found upregulated upon stress or hypoxic conditions, and its encoding gene was under STAT3 transcriptional control [53]. Furthermore, both STAT3 and HIF-1α are responsible for the metabolic shift toward aerobic glycolysis, known as Warburg effect, which is typical of highly aggressive tumours [54].

**Figure 6:**
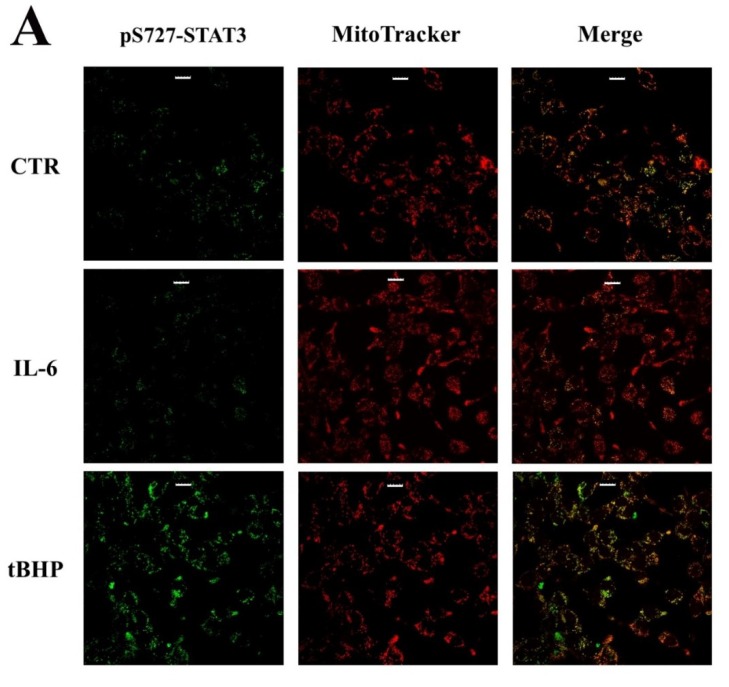

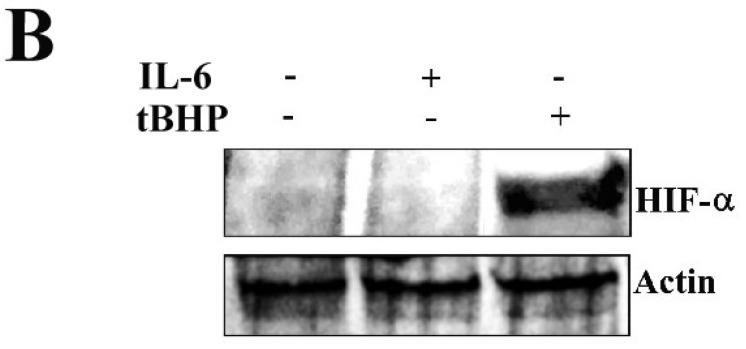
The p-S727 PTM drives the STAT3 non-canonical pathway under oxidative-stress conditions. (**A**) STAT3 mitochondrial localization by immunofluorescence on LNCaP cells both untreated and treated with IL-6 and tBHP. Scale bar 20 µm. (**B**) Western blotting analysis of HIF-1α protein as a sensor of oxidative stress in LNCaP cells both untreated and treated with IL-6 and tBHP.

On the basis of these premises, it is conceivable to hypothesize the involvement of STAT3 phosphorylation at S727 in the development of a more aggressive PCa. Thus, the mitochondrial localization of STAT3 and the expression level of HIF-1α were verified through experiments carried out in LNCaP cells in the same experimental conditions as mentioned above. Mitochondrial STAT3 localization, investigated by immunofluorescence analysis, was detected only in LNCaP cells treated with tBHP (Figure 6A). At the same time, western blotting analysis showed HIF-1α overexpression under oxidative-stress conditions (Figure 6B).

**Figure 7:**
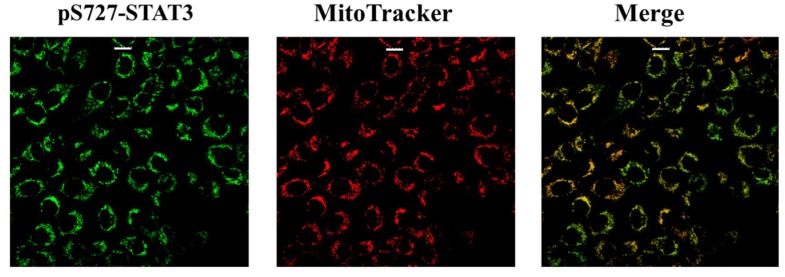
STAT3 mitochondrial localization by immunofluorescence on DU-145 cells. Scale bar 20 µm.

The generation of stressing conditions in LNCaP cells induced by tBHP treatment are highlighted by the increase in ROS level (Table 1). The more aggressive DU-145 cell line, which could be characterized by constitutive oxidative-stress conditions more typical of tumors at advanced clinical phases, was used to compare the results obtained on LNCaP cells. The higher ROS level found in un-stimulated DU-145 cells compared to un-stimulated LNCaP ones was in agreement with the above assumption (Table 1). This is further supported by the constitutive mitochondrial localization of STAT3 in DU-145 cells, as shown in Figure 7. Additionally, after tBHP treatment, ROS levels of LNCaP cells matched the basal amounts detected in DU-145 cells (Table 1). It should be considered that in DU-145 cells there is a mutation in the GSTM2 gene which codes a protein important for ROS elimination COSMIC (https://cancer.sanger.ac.uk/cosmic), and this could partially account the higher ROS level observed in this cell line.

These results seem in accordance with our assumption and are consistent with previous literature data, which found a correlation between an increase in ROS and HIF-1α levels and pS727-STAT3 mitochondrial localization.

### 2.4. STAT3 PTMs in DU-145 Cells

To further reinforce the hypothesis that STAT3 phosphorylation at S727 and glutathionylation may represent a marker for advanced PCa, the analysis of the STAT3 PTM profile was also carried out on DU-145 cells, corresponding to a more aggressive form of PCa.

**Figure 8:**
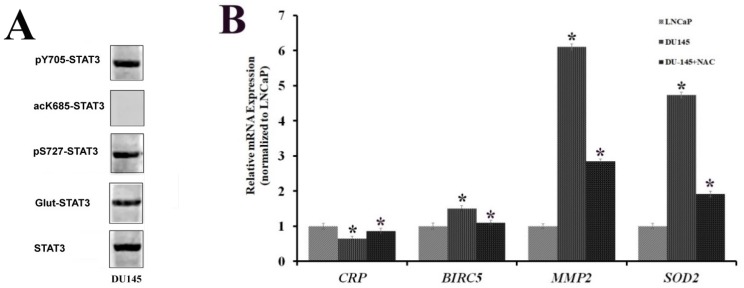
(**A**) Western blotting analysis of STAT3 PTMs on cellular extracts from DU-145 cells. (**B**) Real-time PCR analysis and relative expression of STAT3-target genes, representative of inflammation and oxidative stress in LNCaP cells and in DU-145 cells both untreated and treated for 8 h with 10 μM *N*-acetyl cysteine (NAC). Statistically significant differences (*p* < 0.05) between DU-145 cells both untreated and treated with NAC are marked by *, and values have been normalized to untreated LNCaP.

Previous results suggested the involvement of STAT3 PTMs in different environmental changes induced in LNCaP cells. Since several studies provided evidences that oxidative-stress conditions are predominantly characteristic of advanced stages of PCa, it is conceivable to assume that in a more aggressive prostate cell line, such as DU-145, similar features might be exhibited, as it is generally considered a cellular model representative of an aggressive PCa form. Thus, the STAT3 PTM profile was examined on DU-145 cells by western blot analysis. While STAT3 appeared constitutively glutathionylated as well as phosphorylated at both Y705 and S727 residues, no acetylation at K685 was detectable (Figure 8A).

These results are in accordance with the oxidative-stress conditions induced in LNCaP after tBHP treatment. To further validate this hypothesis, RT-qPCR was performed, analyzing the mRNA levels of the STAT3-specific genes already tested for LNCaP. The results showed the overexpression of *SOD2* and *MMP2* and the down-expression of *CRP* genes in DU-145 compared to untreated LNCaP. Treatment of DU-145 cells with the ROS scavenger *N*-acetylcysteine reduces the expression level of STAT3 target genes to a value comparable to an LNCaP profile (Figure 8B).

**Figure 9:**
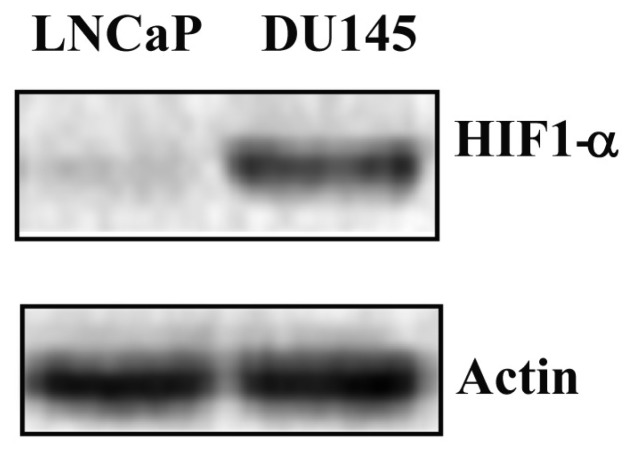
Western blotting analysis of HIF-1α protein expression level in LNCaP and DU-145 cell lines.

These outcomes corresponded with the transcriptional program of STAT3 upon oxidative-stress conditions in LNCaP cells. In fact, the expression profile of tested genes in DU-145 cells followed the same trend as LNCaP cells treated with tBHP. Moreover, the STAT3 PTM profile in DU-145 cells was comparable to that obtained in a previous study on prostate FFPE tissues with higher Gleason score [28]. These results confirmed the involvement of STAT3 PTMs in the progression of PCa into a more aggressive form. For this reason, HIF-1α was analysed by western blotting in both LNCaP and DU-145 cells. As shown in Figure 9, HIF-1α is constitutively present in DU-145 cells, and its expression level is comparable to that observed in LNCaP cells after treatment with tBHP.

These results highlighted the role of STAT3 and its PTMs as drivers in the progression of PCa. In addition, recent studies provide evidence that STAT3 phosphorylated on residues pY705 and/or pS727 is related to HIF-1α expression [51].

## 3. Discussion and Conclusions

The adding of specific chemical groups to proteins often affects their structural dynamism and plasticity, thus leading to their biological versatility. STAT3 represents an example of how unusual PTMs can influence its cellular localization, thereby affecting its activity and modulating both its canonical and non-canonical pathways. STAT3 is a well-known oncoprotein and can mediate the activation of several cytoplasmic signaling cascades upon its phosphorylation at Y705 residue. In particular, this PTM induces a conformational change in STAT3 protein, which undergoes homodimerization and then translocates into the nucleus. Once there, the protein can exert its role as a transcription factor by controlling the expression levels of specific target genes.

In vitro studies performed on less-aggressive LNCaP and more-aggressive DU-145 cell lines put the spotlight not only on STAT3 phosphorylation at Y705, which is associated with STAT3 transcriptional functions, but also on other modifications that diversify STAT3 activities. In fact, the phosphorylation at S727, the glutathionylation at C328/C542 and the acetylation at K685 are widely described in literature and seem related to specific STAT3 biological behaviors.

As highlighted in a previous study [28] carried out on a panel of prostate cancer FFPE tissues at different Gleason scores, STAT3 PTMs could play a not-negligible part in PCa progression. The Gleason score is a grading system that identifies the aggressiveness of prostate cancer based on its morphological characteristics. In particular, prostate cancer with higher Gleason scores is more aggressive and is associated with a worse prognosis compared with lower Gleason scores.

Our study found that the distribution of specific STAT3 PTMs in prostate tissues at different Gleason scores followed a specific expression program. Indeed, while STAT3 acetylation at K685 was observed in tissues with a value of Gleason score 6, characterized by an overall inflammatory condition, STAT3 glutathionylation and phosphorylation at S727 were present in Gleason score 9, where the oxidative stress is predominant. These PTMs reflect STAT3 transcriptional programs under particular cellular conditions.

In the present work, the existence of a link between PTMs and specific STAT3-mediated pathways was investigated in LNCaP and DU-145 cells performing experiments that simulated inflammatory and oxidative-stress conditions. Cellular studies confirmed the stimulus-specificity of STAT3 PTMs. The phosphorylation at Y705 represented a common denominator of all STAT3 signaling. In addition, STAT3 was acetylated in response to cytokines, whereas its glutathionylation and phosphorylation at S727 occurred upon tBHP treatment. Thus, different PTMs may represent dissimilar intracellular conditions. Although no evidence was reported about the transcriptional activity of STAT3 after its glutathionylation, this PTM could act as molecular switch addressing the activation of STAT3 through its non-canonical pathway in response to oxidative stress. Obtained results also provided evidence of the interplay between STAT3 PTMs and specific protein interactors, such as P300 and APE1/Ref-1. In particular, the presence of P300 as a STAT3 co-interactor was identified in samples treated with IL-6, while APE1/Ref-1 was detected in conjunction with STAT3 activation under oxidative stress, which appears correlated to glutathionylation. In fact, immunocomplexes obtained from LNCaP cells treated with tBHP and DTT did not show the presence of APE1/Ref-1 protein.

In accordance with these outcomes, mRNA levels of STAT3-target genes seemed to follow the distribution of STAT3 PTMs. In fact, the *CRP* gene was found overexpressed concomitantly with STAT3 acetylation and the specificity of this response was further demonstrated by using an inhibitor (C646) that switched off the signal. On the contrary, *BIRC5* has resulted under Glut-STAT3 control and its level of expression increased upon tBHP treatment, as previously described by Butturini et al. [24]. Finally, *SOD2* and *MMP-2* were shown to be overexpressed at the same time as STAT3 glutathionylation and phosphorylation at S727. DTT, as a suppressant of glutathionylation, seemed not to affect their expression profiles, probably because these two genes could both be regulated by the STAT3 non-canonical pathway.

It is conceivable to hypothesize that the phosphorylation at Y705, the glutathionylation and the acetylation could affect STAT3 activity as a transcription factor. In addition, it is worthwhile to underline that pS727-STAT3, localized into mitochondria (Figure 6A), could also be involved in the metabolic reprogramming that drives the aggressive features of cancer. Experiments performed on the more-aggressive DU-145 cell line revealed a constitutively STAT3 activation by means of its phosphorylation at residue Y705, as well as its glutathionylation and phosphorylation at S727 [24,55]. The STAT3 PTM profile in DU-145 cells, as well as its transcriptional program, is comparable to that observed in LNCaP cells under oxidative-stress conditions.

Obtained results on cellular models confirmed the relationship between STAT3 PTMs and cellular conditions, thereby reinforcing the hypothesis that PTMs can drive intracellular responses through STAT3-mediated signaling pathways. Thus, these evidence identifies STAT3 PTMs and STAT3 modulators as suitable markers or targets for PCa prevention, diagnosis and therapy.

## 4. Material and Methods

### 4.1. Cell Culture

Human prostate cancer cell lines LNCaP and DU-145 were obtained from the American Type Culture Collection (ATCC). Cells were grown to 80% confluence at 37 °C in 5% CO_2_ in the appropriate culture medium (RPMI 1640 (Sigma-Aldrich, Milano, Italy) or MEME (Sigma-Aldrich)) and supplemented with 1% sodium pyruvate, 10% fetal bovine serum, 2 mM glutamine, 100 μg/mL streptomycin and 100 U/mL penicillin.

Cells were tested with interleukin-6 (IL-6) (RELIATech, #200-030) at a final concentration of 50 ng/mL for 15 min, 75 µM tert-butyl hydroperoxyde (tBHP) for 30 min (Sigma Aldrich, 458139) and 100 µM H_2_O_2_ for 15 min (Sigma Aldrich, 7722-84-1). C646 (Sigma Aldrich, SML0002) and dithiothreitol (DTT) (Sigma Aldrich, 3483-12-3) were both used at a final concentration of 100 µM. Cells were pre-treated with C646 overnight, whereas they were treated with DTT for 1 h after tBHP stimulation.

### 4.2. Proteins Extraction and Immunoblotting

Cells cultured on 6-well plates were scraped, harvested by centrifugation and washed in PBS. To obtain total protein extracts, cell pellets were immediately lysed in buffer containing 2% SDS, 20 mM Tris-hydrochloride pH 7.4, 2 M urea, 10% glycerol, 2 mM sodium orthovanadate, 10 mM DTT and a protease inhibitor cocktail diluted 1:100 (Sigma-Aldrich).

Nuclei were obtained from cell pellets using a hypotonic buffer (10 mM HEPES, 10 mM KCl, 1.5 mM MgCl2, 0.5 mM DTT) added with 0.05% Triton-X, 2 mM sodium orthovanadate and a protease inhibitor cocktail diluted 1:100 (Sigma-Aldrich). Thus, nuclei were harvested by centrifugation and washed in hypotonic buffer, and nuclear protein extracts were obtained as described above for total protein extracts.

Proteins were resolved by SDS-PAGE 10% TGX FastCast Acrylamide gel (BioRad, Segrate, Italy) and transferred on polyvinylidene fluoride PVDF membranes (BioRad, Segrate, Italy) using the Trans-Blot Turbo Transfer System (BioRad). The membranes were blocked with 3% *w/v* Albumin Bovine Serum BSA (Carl Roth, Milano, Italy, CAS No. 0163.4) in Tris-buffered saline, and incubated with a specific primary antibody for 1 h. Subsequently, membranes were washed three times in BSA 2% *w/v* in TBS, and then incubated for an additional hour with the appropriate alkaline phosphatase-conjugated secondary antibody (Jackson ImmunoResearch, Pero, Italy). The alkaline phosphatase signal was detected with BCIP/NBT reagents (Carl Roth, Milano, Italy, CAS No. 298-83-9 and 6578-06-9). β-actin (total extracts) or lamin (nuclear extracts) were used as a normalization protein.

The immunoblotting detection was carried out using monoclonal anti-STAT3 (Cell Signaling D3Z2G, antibody dilution 1:1000), monoclonal anti-pY^705^STAT3 (Cell Signaling D3A7, antibody dilution 1:2000), polyclonal anti-pS^727^STAT3 (Sigma-Aldrich, SAB4300034, antibody dilution 1:1000), polyclonal anti-AcK^685^STAT3 (Cell Signaling 2523s, antibody dilution 1:1000), monoclonal anti-p300 (Cell Signaling D8Z4E, antibody dilution 1:1000), polyclonal anti-Ape/Ref-1 (Cell Signaling, 4128, antibody dilution 1:1000), polyclonal anti-β-actin (Cell signalling, 4967, antibody dilution 1:1000). Each experiment was replicated at least three times.

### 4.3. Analysis of S-Glutathionylated STAT3

Cells were lysed at 4 °C in RIPA buffer (20 mM Tris HCl, pH 8.0, 150 mM NaCl, 1% Nonidet P-40, 1 mM EDTA, 10% glycerol, 100 mM NaF, 1 mM Na_3_VO_4_ and protease cocktail inhibitor). Equal amounts of proteins from the clarified cell lysates were incubated overnight at 4 °C with anti-STAT3 antibody (cell signaling) immobilized on IgG rabbit magnetic beads (Dynabeads, Invitrogen Thermo Fisher Scientific, Rodano, Italy). The immune complexes were eluted in a non-reducing sample buffer (62.5 mM Tris HCl, pH 6.8, 10% glycerol, 5% SDS, 0.05% bromophenol blue), analyzed by immunoblotting, with monoclonal Anti-Glutathione (Abcam [D8] ab19534 antibody dilution 1:1000) [56].

### 4.4. Reactive Oxygen Species (ROS) Detection

Reactive oxygen species (ROS) generated by stressing cells with 75 µM tBHP were quantified using the CellROX Green Flow Cytometry Assay Kit (Thermo Fisher Scientific, Rodano, Italy, C10492) following the manufacturer’s instructions. Samples were analyzed by a BD Accuri C6 flow cytometer (BD Biosciences).

### 4.5. Extraction of RNA and RT-qPCR

Total RNA was extracted from cells using TRIzol reagents (Immunological Science) in accordance with the manufacturer’s instructions. RNA was quantified spectrophotometrically, and its quality was assessed by 1% agarose gel electrophoresis and staining with ethidium bromide. The reverse transcription was carried out with Super Script II R-Transcriptase (FS-RT-3022, Fisher Molecular Biology). Gene expression was evaluated with specific primers for *SOD2, BIRC-5, c-MYC, MMP-2* and *CRP* (RT^2^-qPCR Primer Assays: Human CRP (NM_000567). Cat.no PPH02632A, Human BIRC5 (NM_001012270) Cat.no PPH00271E, Human MMP2 (NM_001127891) Cat.no PPH00151B and Human SOD2 (NM_000636) Cat.no PPH01716B (all from Qiagen S.r.l., Milano, Italy) were evaluated using the CFX Connect Real-Time PCR Detection System (BioRad Laboratories, Segrate, Italy) with a SYBR green fluorophore-based real-time reaction (Brilliant SYBR Green QPCR Master Mix, Thermo Fisher Scientific). Expression data were analyzed using CFX Manager Real Time PCR Detection System Software, version 3.1 (BioRad).

### 4.6. Immunofluorescence

Cultured LNCaP and DU-145 cells were grown on coverslips, and LNCaP were treated with IL-6 or tBHP in the same aforementioned conditions. After the stimulation, cells were washed with PBS, fixed with 4% formaldehyde for 15 min and then rinsed with PBS. Cell were then permeabilized with cold MeOH (−20 °C) for 5 min. After washing three times with PBS, cells were blocked overnight with 3% *w/v* BSA (Sigma-Aldrich) in PBS. Fixed cells were processed for immunofluorescence staining to detect the localization of pS^727^-STAT3 (Sigma-Aldrich, SAB4300034, antibody dilution 1:200) using a specific primary antibody diluted in PBS containing 2% *w/v* BSA for 1 h. Following three washes with PBS added with 0.05% Triton and 2% *w/v* BSA (PBS-T), cells were incubated for 1 h in the darkness with a Fluorescein isothiocyanate FITC-conjugated secondary antibody (Jackson Immunoresearch, dilution 1:800). Cell nuclei were counterstained with 100 ng/mL Hoechst 33258 for 15 min. After washing with PBS-T, coverslips were mounted on glass microscopy slides with Duolink Mounting Medium and examined with a confocal fluorescence microscope (ZEISS LSM510META) with magnification using 60x oil immersion objective lens. Images were acquired and analysed by ZEN 215 software (ZEISS, Germany).

### 4.7. Mitochondria Staining

Mitochondria were stained with MitoTracker Orange CMTMRos (Invitrogen, M7510) before fixation. MitoTracker 1 mM (DMSO stock solution) was diluted to a final concentration of 190 nM in serum-free culture media in accordance with the manufacturer’s instruction. Cells were incubated for 30 min with the mitochondrial dye and then washed three times in serum-free culture media before fixation and immunofluorescence analysis.

### 4.8. Chromatin Immunoprecipitation (ChIP) and Co-Immunoprecipitation Assays

DNA-protein and protein–protein cross-linked complexes were formed in LNCaP and DU-145 cell lines, with or without stimulation (IL-6/tBHP), using 1% formaldehyde (5 min at 37 °C) according to method described in Eufemi et al. [57]. The immunoprecipitation procedure was carried out by anti-STAT3 antibody immobilized on IgG rabbit magnetic beads (Dynabeads, Invitrogen). The mock sample was immunopurified with preimmune IgGs. The DNA was then purified by phenol-chloroform extraction and ethanol precipitation and amplified by PCR analysis. The amount of immunoprecipitated DNA subjected to amplification was measured by means of a QUBIT Fluorometer (Invitrogen) following the manufacturer’s instructions. Primers used for gene promoters were:

*Crp*: For 5′-GAGTTTGTAATAAATAAC-3′ (TSS-172/-155)
  Rev 5′-CACTATGTAAATAATTTTC-3′ (TSS-85/-67),
*Birc5*: For 5′-GTTGCAGTGAGCTGAGATC-3′ (TSS-1114/-1095)
  Rev 5′-GTGCCTCCACTGTCTTTTTC-3′ (TSS-917/937),
*Mmp2*: For 5′-CAAGATGGAGTCGCTCTG-3′ (TSS -650/-633)
  Rev 5′-GTAAGCCTTAACTTGGCCTC-3′ (TSS-580/599),
*Sod2*: For 5′-GTGGGTGTCCAAGAACTGT-3′ (TSS-748/730)
  Rev 5′-GTACTCTTGCGCCGTACC-3′ (TSS-643/-626).

Proteins were purified from immunocomplexes and identified by immunoblotting.

### 4.9. Statistical Analysis

The repeatability of results was confirmed by performing all experiments at least three times. The obtained values are presented as mean and standard deviation. Statistical analysis was performed with GraphPad Prisma software using a Student’s *t*-test.

## Figures and Tables

**Table 1:** Quantification of ROS in untreated LNCaP and DU-145 cells, and in LNCaP cells treated for 30 min with 75 µM tBHP.

Cell Line	Cell ROS (Mean Fluorescence)
LNCaP	32,435.25
LNCaP + tBHP	45,390.62
DU-145	41,480.41
